# Effect of Fc Receptor Genetic Diversity on HIV-1 Disease Pathogenesis

**DOI:** 10.3389/fimmu.2019.00970

**Published:** 2019-05-09

**Authors:** Daniel E. Geraghty, Christian W. Thorball, Jacques Fellay, Rasmi Thomas

**Affiliations:** ^1^Clinical Research Division, Fred Hutchinson Cancer Research Center, Seattle, WA, United States; ^2^School of Life Sciences, École Polytechnique Fédérale de Lausanne, Lausanne, Switzerland; ^3^Precision Medicine Unit, Lausanne University Hospital and University of Lausanne, Lausanne, Switzerland; ^4^U. S. Military HIV Research Program, Walter Reed Army Institute of Research, Silver Spring, MD, United States; ^5^Henry M. Jackson Foundation for the Advancement of Military Medicine, Bethesda, MD, United States

**Keywords:** next-generation sequencing, polymorphism, disease association, Fc receptors, HIV-1

## Abstract

Fc receptor (FcR) genes collectively have copy number and allelic polymorphisms that have been implicated in multiple inflammatory and autoimmune diseases. This variation might also be involved in etiology of infectious diseases. The protective role of Fc-mediated antibody-function in HIV-1 immunity has led to the investigation of specific polymorphisms in FcR genes on acquisition, disease progression, and vaccine efficacy in natural history cohorts. The purpose of this review is not only to explore these known HIV-1 host genetic associations, but also to re-evaluate them in the context of genome-wide data. In the current era of effective anti-retroviral therapy, the potential impact of such variation on post-treatment cohorts cannot go unheeded and is discussed here in the light of current findings. Specific polymorphisms associating with HIV-1 pathogenesis have previously been genotyped by assays that captured only the single-nucleotide polymorphism (SNP) of interest without relative information of neighboring variants. With recent technological advances, variation within these genes can now be characterized using next-generation sequencing, allowing precise annotation of the whole chromosomal region. We herein also discuss updates in the annotation of common FcR variants that have been previously associated with HIV-1 pathogenesis.

## Introduction

Fc receptors comprise a class of cell surface receptors expressed on various hematopoietic cells that bind to the Fc portion of antibodies to form immune complexes and recruit the complement and/or effector system to defend the body against pathogens. The Fc receptors are classified based on their binding to the Fc domain of immunoglobulin (Ig). The most abundant Ig in serum is IgG which can bind to different classes of FcγR. Other types of FcR including FcεR, Fcα/μR, and FcαR1 are receptors for other Ig classes such as IgE, IgM, and IgA.

More recently vaccine studies in infectious diseases point to a critical role of non-neutralizing antibody functions, which is the ability of an antibody to interact with other immune components and effector cells via their Fc portions to mediate killing or control of the pathogen. These mechanisms include, but are not limited to, antibody dependent cellular cytotoxicity (ADCC), antibody-dependent cell-mediated virus inhibition (ADCVI), antibody dependent cellular phagocytosis (ADCP), and antibody dependent complement deposition (ADCD) (1, 2). These functions are mediated by three distinct classes of FcγRs that are expressed on most human immune cells, with varying levels of expression dependent on cell type such as monocytes, macrophages, natural killer cells, eosinophils, neutrophils, B cells but not on T cells (3). These receptors include FcγRI (CD64), FcγRIIa/b/c (CD32), and FcγRIIIa/b (CD16), which bind the different IgG subclasses with varying proficiency, and can cause either activation or inhibition of the effector cell.

Polymorphisms in the FcγR have been shown to affect binding affinity to the Fc region of IgG and can trigger a range of effector and immunoregulatory functions. Such variation has been shown to play a crucial role in the pathogenesis of a range of chronic inflammatory and autoimmune diseases, as well as susceptibility to infectious pathogens (4, 5). An overview of FcγR biology has been recently summarized and so our focus for this review will be to evaluate the effect of genetic variation in the human Fcγ receptors and their role specifically in HIV-1 disease pathogenesis (6). Although there is evidence that the neonatal Fc receptor (FcRn), an MHC class I-related molecule expressed on many cells, functions in HIV-1 vaccination and infection (7, 8), no significant genetic variation has been identified for this locus, and we have not included it in this review.

## Fcγ Receptor Genetic Diversity

**Figure 1:**
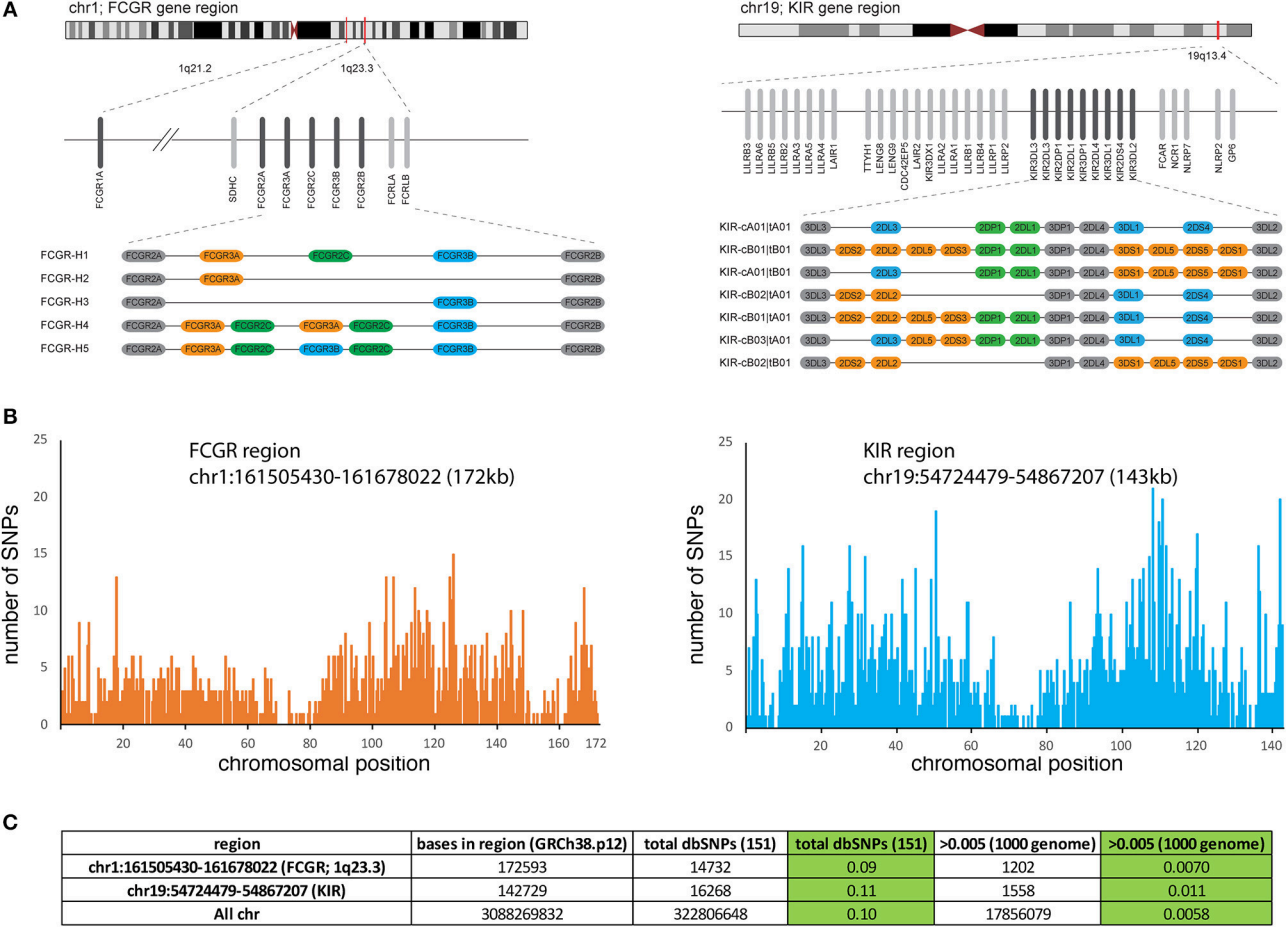
Structure and polymorphism of the FCGR genes. **(A)** Approximate chromosomal locations of the FCGR gene complex containing 5 FCGR genes and the FCGR1A gene located at ~12 Mb centromeric to the FCGR cluster. In comparison, the KIR gene family is illustrated immediately to the right including adjacent genes (FCAR) and gene clusters. For both FCGR and KIR, alternative haplotypes identified in populations are illustrated with the colored bars depicting genes present in a subset of haplotypes and shaded bars depicting genes present in all haplotypes (framework genes). Figure depicting KIR has been reproduced with permission from the Oxford University Press (9). **(B)** The plots show the abundance of SNPs at each position of the indicated regions from chr1 and chr19, using data derived from the UCSC genome browser. **(C)** Approximate number of SNPs in both regions are listed including a summary from all chromosomes for comparison.

The Fcγ receptors are encoded by the FCGR genes located on chromosome 1 in humans, including five FCGRs in a tandem arrangement within ~200 kb of genomic sequence (Figure 1A). A sixth gene, FCGR1A, is located ~12 Mb distant from the five gene cluster. Genetic variation at the FCGR gene cluster bears similarity to the Killer Ig-like receptor (KIR) region which is shown in comparison to emphasize both the types and extent of copy number and allelic variation (Figures 1A,B) (9). Like KIR, the genes in the FCGR cluster are arranged in haplotypes containing both invariant framework and copy number variant genes. An examination of total nucleotide variation in FCGR from a recent genome build indicates extensive depths of SNP variation, similar in overall extent to KIR and the vast majority of which has not been functionally characterized (Figures 1B,C). Both gene families encode receptors for other central components of the immune response (KIR and MHC class I; FCGR and IgG constant domains) placing them in distinct roles but perhaps of equivalent importance in investigations of host genetics and its relationship to immune function. Given the parallels in significance and the similar physical characteristics of both copy number variation and allelic polymorphism, a major difference is that the allelic variations for FCGR genes have been less examined, curated and annotated. This review in part is attempting to address this deficit as an organizing framework of characterized variation possibly guided by established methods for structural and allelic annotation as currently employed for the KIR system (10).

In the FCGR family of six genes, several nonsynonymous single nucleotide polymorphisms (SNP), SNPs encoding altered splice sites, and copy number variants (CNVs) encoding addition, or deletion of one or more gene have been functionally characterized (Figure 1, Table 1). Variation in the FCGR1 gene is limited, with the most frequent minor allele characterized at < 4%, but there is considerable diversity in the other FCGR genes. The most studied SNPs in the FCGR genes over the past two decades have centered on nonsynonymous substitutions that contribute to differential binding affinity for subclasses of IgG. FCGR2A has two allele variants encoding arginine or histidine at amino acid position 166 (rs1801274), with the latter resulting in a higher affinity for IgG1, and IgG2 (11–13). FCGR3A also has two common allele variants differing by a single SNP, altering codon 176 from phenylalanine to valine (rs396991) resulting in a higher affinity for IgG1, IgG2, IgG3, and IgG4 for the 176V variant (11, 12, 14). This stronger binding affinity is associated with functional capacity of the receptor in different experimental and clinical contexts (15–17). Two adjacent nonsynonymous SNPs in FCGR2A (together altering codon 63 from Q to W; rs201218628) have been studied, although their functional consequence is less clear, and their frequency is rare (18). FCGR2B has an isoleucine to threonine change at position 232 (rs1050501), which alters the transmembrane region, with the 232T allele inhibiting the association of FCGR2B with lipid rafts in a human B cell line measuring downstream function (19). The frequency of this alteration is low at ~1% in Caucasians, and while more prevalent among African Americans and Asians (5–11%) has been less studied.

**Table 1:** Characterization of variation in the Fc Receptor genes.

**Chr. location (GRCh38.p7)**	**dbSNP rs#**	**MAF**	**Function**	**RefSeqGene (genomic DNA)**	**RefSeqGene (mRNA)**	**SNP variants^**a**^**	**RefSeqGene (Protein)**	**Amino acid change**
**FCGR2A**				NG_012066	NM_021642.3		NP_067674.2	
161506414-161506415	rs201218628	0.006	Missense	6000_6001	184_185	CA>TG	62	Gln>Trp
161509955	rs1801274^d^	0.442	Missense	9541	497	A>G	166	His>Arg
161510070	rs150311303	0.008	Insertion	9657_9658	613_614	insTTC	205	Gln>GlnLeu
161510859	rs11810143	0.055	synonymous	10445	642	A>G	214	Pro>Pro
161510928	rs140474146	0.009	Synonymous	10514	711	G>A	237	Leu>Leu
161518073	rs12029217	0.121	Synonymous	17659	876	C>T	292	Pro>Pro
161518091	rs6694457	0.026	Synonymous	17677	894	T>C	298	Asp>Asp
**FCGR2B**				NG_023318	NM_004001.4		NP_003992.3	
161671501	rs148030870	0.010	synonymous	13387	243	C>T	81	Ser>Ser
161671594	rs6665610	0.138	Synonymous	13480	336	G>A	112	Thr>Thr
161671618	rs367584808	0.005	Synonymous	13504	360	C>A	120	Leu>Lue
161672984	rs200112434	0.109	Missense	14870	401	T>G	134	Val>Gly
161673192	rs2298022	0.029	Synonymous	15078	609	G>A	203	Thr>Thr
161673195	rs182968886	0.110	Synonymous	15081	612	G>A	204	Leu>Lue
161674008	rs1050501	0.186	Missense	15894	695	T>C	232	Ile>Thr
161675262	rs28651835	0.032	Missense	17148	766	C>T	256	Pro>Ser
**FCGR2C**				NG_011982	NM_201563.5^b^		NP_963857.3	
161589466	rs114945036	0.245	Intron	13128		C>T		
161589597	rs759550223	0.002	Nonsense	13259	169	C>T	57	Gln>^*^Ter
161589781	rs138747765	0.194	Missense	13443	353	C>T	118	Thr>Ile
161589930	rs78603008	0.195	Intron	13592		G>A		
161591153	rs370748254	0.134	Missense	14815	401	T>G	134	Val>Gly
161591361	rs74341264	0.199	Synonymous	15023	609	G>A	203	Thr>Thr
161591366	rs76016754	0.034	Missense	15028	614	A>T	205	Tyr>Phe
161599629	rs430178	0.259	Splice acceptor	23291		C>G		
161599779	rs138731942	0.075	Synonymous	23441	948	C>T	316	Asn>Asn
**FCGR3A**				NG_009066	NM_001127593.1		NP_001121065.1	
161543083	rs115866423	0.006	Missense	12541	694	A>T	232	Asn>Tyr
161544752	rs396991^d^	0.351	Missense	10872	526	T>G	176	Phe>Val
161548509	rs150808747	0.012	Synonymous	7115	231	C>T	77	Asp>Asp
161548524	rs114535887	0.019	Synonymous	7100	216	G>A	72	Ser>Ser
161548543	rs10127939	0.039	Missense	7081	197	T>G/T>A	66	Leu>Arg/Leu>His
**FCGR3B**				NG_032926	NM_000570.4		NP_000561.3	
161626224	rs71632957	0.022	Synonymous	10740	498	T>C	166	Asp>Asp
161626242	rs114169903	0.025	Synonymous	10722	480	A>G	160	Pro>Pro
161629781	rs2290834	0.447	Missense	7183	316	A>G	106	Ile>Val
161629800	rs368410676	0.023	Synonymous	7164	297	G>T	99	Pro>Pro
161629853	rs147574249	0.284	Missense	7111	244	A>G	82	Asn>Asp
161629864	rs5030738	0.083	Missense	7100	233	C>A	78	Ala>Asp
161629903	rs448740	0.467	Missense	7061	194	A>G	65	Asn>Ser
161629983	rs527909462	0.135	Synonymous	6981	114	T>C	38	Leu>Leu
161629989	rs200688856	0.128	Missense	6975	108	C>G	36	Ser>Arg
**FCGR1A**				NG_007578	NM_000566.3		NP_000557.1	
149784005	rs138447715	0.034	Missense	6274	55	A>G	19	Thr>Ala
149784064	rs80039899	0.017	Synonymous	6333	114	C>T	38	Thr>Thr
149784065	rs7531523	0.005	Missense	6334	115	G>A	39	Val>Ile
149784139	rs149926813	0.005	Synonymous	6408	189	T>C	63	Thr>Thr
149784147	rs144081076	0.004	Missense	6416	197	C>T	66	Ser>Leu
149784224	rs74315310	0.004	Nonsense	6493	274	C>T	92	Arg>^*^Ter
149788436	rs138510822	0.008	Synonymous	10705	378	G>A	126	Ala>Ala
149790290	rs587727639	0.008	Missense	12559	796	G>A	266	Asp>Asn
**FCAR**				Na^c^	NM_002000.3		NP_001991.1	
54885265	rs61735068	0.006	Missense		101	A>C	34	Lys>Thr
54885488	rs1865096	0.261	Synonymous		324	G>A	108	Arg>Arg
54885501	rs11666735	0.055	Missense		337	G>A	113	Asp>Asn
54888276	rs61735069	0.042	synonymous		631	T>C	211	Leu>Leu
54889758	rs77103719	0.006	Synonymous		759	G>A	253	Thr>Thr
54889796	rs61735070	0.006	Missense		797	C>T	266	Pro>Leu
54889804	rs16986050	0.155	Missense		805	A>G	269	Ser>Gly

a *Only SNP variants with a MAF of >0.01 are shown*.

b *The status of FCGR2C has been recently changed from gene to pseudogene in genbank*.

c *No unique genome reference sequence has been defined for FCAR in genbank at this point*.

d *rs1801274 and rs396991 have been commonly referenced in the literature as FCGR2A-131R/H and FCGR3A-158F/V, respectively*.

A major studied polymorphism in the FCGR2C gene is a SNP in exon 3 (rs759550223) that encodes a glutamine or a stop codon resulting in the presence or absence of protein expression (20–22). The frequency of the minor allele varies between populations, and studies have suggested it is expressed on NK cells and is capable of inducing ADCC after receptor cross-linking on purified NK cells as measured by their ability to lyse the target P815 cell line (20, 23). In addition, both alleles have been associated with both null and surface expression on NK cells as measured by anti-FCGR2B/C specific mAb 2B6 (22). However, there may be some confusion regarding this SNP as it is identical to rs10917661, which is assigned to FCGR2B, in a reference SNP identification (rs id) segment where the two genes have identical sequences except at the variant position. Sequence identity between FCGR2B and FCGR2C may lead to incorrect assignment of SNPs to these two loci. Also, rs759550223 has a very low minor allele frequency defined in the SNP database (dbSNP), and the minor allele assigned is identical to the FCGR2B-derived sequence, suggesting the possibility that the SNP has been falsely generated by a combination of variants between two distinct loci. FCGR2C has been previously reported to have arisen from an unequal recombination between the FCGR2A and FCGR2B genes, and encoded a functional molecule that exhibited differential expression in natural killer cells (21, 24, 25). However, FCGR2C is also classified as a gene/pseudogene in the NCBI gene database. These inconsistencies further emphasize the need of validation of the FCGR genes by a combination of methods such as next generation sequencing (NGS) technologies as discussed below, in addition to precise curating of allelic variation and flow cytometry phenotyping.

A triallelic nonsynonymous SNP at codon 66 (66R, 66L, 66H; rs10127939) of the FCGR3A gene has also been found to affect affinity for immune complexes (ICs), with the FCGR3A-66R and 66H alleles exhibiting higher affinity (26). Other nonsynonymous SNPs have been identified but none have been characterized functionally or in association analyses. FCRG3B polymorphisms were first described as the human neutrophil antigen (HNA)-1 system (27, 28). The three major HNA-1 variants have differential affinity for IgG1 and IgG3, with the higher affinity HNA-1a and lower affinity HNA-1b differing at 4 nonsynonymous codon positions (rs2290834, rs200688856, rs448740, rs147574249). Consistent with this differential affinity, phagocytosis was lower with HNA-1b through analysis of antibacterial IgG subclass antibodies and with IgG1 and IgG3 anti-Rhesus D (29, 30). A third isoform, termed HNA-1c, of unknown function is identical to the HNA-1b isoform except at the rs5030738 polymorphic site, where it encodes an asparagine rather than alanine residue (31). Other variants of the HNA-1 antigen system have also been described but to date no functional or association studies interrogating them have been reported (32, 33).

CNV is a hallmark of multicopy gene family genomic regions, including notably among them, those encoding immune response genes (34). In the FCGR region, CNVs include at least five haplotypes with varying combinations of deletions and duplications of the FCGR2C, FCGR3A, and FCGR3B genes, flanked by the invariant framework FCGR2A and FCGR2B genes (Figure 1A, *left*) (34–36). FCGR-H1 forms the most common among these haplotypes, containing the five loci, with FCGR-H2 being the most commonly observed CNV [equivalent to CNR1 in Nederer et al. (36)] and FCGR-H3 less prevalent (equivalent to CNR2 or CNR3). Although variants FCGR-H4 and -H5 have not been explicitly described, they form predicted reciprocal structures of the H2 and H3 deletion variants (34). Individuals with FCGR gene copy numbers that may be consistent with those structures have been described (22, 34–38). As discussed above, older genotyping methods focusing on specific regions of the genes may have misassigned SNPs, and the lack of a standard nomenclature of common FGCR coding variants may lead to misinterpretation when comparing different studies. Newer NGS technologies have allowed for updated annotation of the FCGR genes as per the current Human genome database reference hg38. SNPs with a minor allele frequency >0.01 are shown in Table 1.

## FCGR Variation and HIV-1 Disease Pathogenesis

The first report of the effect of polymorphisms in the FCGR genes on HIV-1 disease progression was in two natural history HIV-1 cohorts consisting of anti-retroviral therapy (ART) naïve individuals (39). Since then, functional SNPs in the FCGR2A (rs1801274) and FCGR3A (rs396991) genes that affect binding affinity to the Fc domain of IgG have been evaluated in the context of HIV-1 acquisition, disease progression, and vaccine efficacy. Now that most HIV-1 infected individuals are on ART, there is an opportunity to evaluate disease outcomes after ART initiation. With increased high-throughput sequencing, targeted SNP genotyping is being replaced by whole gene and genome sequencing. This gives the opportunity to evaluate previous host genetic findings in the light of genome wide findings and also examine other SNPs in nearby genes. We will discuss the effects of genetic polymorphisms in the FCGR genes and their impact on HIV-1 disease progression, acquisition, post-ART and vaccine outcomes in the next sections.

### FCGR Polymorphisms and HIV-1 Disease Progression

#### Candidate Gene Studies

Forthal et al. identified an association between the FCGR2A low binding RR (rs1801274) genotype and a faster rate of CD4+ T cell decline and progression to AIDS using samples and data from the Multicenter AIDS Cohort Study (MACS) consisting of more than 500 HIV-1 infected males of mostly Western European ancestry (40). Paradoxically, the same RR genotype was also found to associate with a decreased risk of Pneumocystis jiroveci (carinii) pneumonia, an AIDS defining illness, when compared to the HH genotype in the same cohort. At the functional level, cells from RR homozygous carriers demonstrated less efficient phagocytosis of HIV-1/IgG complexes. There was no association of the FCGR2A genotype with viral load setpoint (spVL), defined as the number of HIV-1 RNA copies/ml in a plasma sample collected 18 months after the first seropositive test. The absence of association of FCGR2A variation with spontaneous viral load control was also confirmed in an HIV-1 seroconverting cohort including 253 Kenyan women, in which the associations with disease progression and CD4+ T cell decline were not replicated (41).

No association was observed by Forthal et al. in the MACS cohort between a specific FCGR3A genotype (rs396991) and spontaneous viral control or disease progression (40). Similarly, Weis et al. did not identify any significant genetic associations of FCGR3A variation with disease progression or spVL in the Kenyan's women cohort (41). There is one report of the VV genotype of FCGR3A being overrepresented in 43 untreated controllers compared to 59 HIV positive progressors on ART (42). However, since the HIV positive progressors were on ART, analyses with measures of spVL or CD4+ T cell counts could not be performed and this finding remains inconclusive.

A more consistent finding has been reported by two independent groups showing the association between the FCGR3A FF genotype and decreased risk of Kaposi's sarcoma (KS) (39, 40). In the first study, FCGR3A genotyping was performed in two small cohorts consisting of 119 and 131 HIV-1 infected males of Western European ancestry. A significant association with protection was identified in each cohort independently and in the combined analysis. Forthal et al. replicated this finding in the MACS cohort. KS is the most frequent malignant condition associated with HIV-1 related immunosuppression, and alterations in the cytokine balance have been suggested to play a critical role in its pathogenesis. Differences in genotype have been shown to alter IgG binding that could influence cytokine levels, with the V allele having higher affinity than the F. The authors concluded that FF homozygous individuals might be at lower risk of KS because of a less vigorous proinflammatory response. The VV genotype has been associated with an increased risk of cryptococcal disease in 164 HIV-1 infected men, again in the MACS cohort (43). Of note, this observation extends beyond HIV-1 infection, because the VV genotype was previously associated with cryptococcal disease in non-HIV-infected individuals in a separate study (44).

#### Genome-Wide Testing

The associations with HIV-1 natural history described above were tested using a candidate gene study design in cohorts with relatively small sample size. The current availability of genome-wide genotyping and sequencing data provides an opportunity to reassess the potential involvement of FCGR variation in HIV-1 disease in larger cohorts, by applying more stringent standards for significance level and including robust population stratification (45). We therefore accessed previously published data generated from cohorts and studies that contributed to the International Collaboration for the Genomics of HIV (ICGH) (46–48) and assessed genetic associations with HIV-1 disease outcomes in the FCGR2A and FCGR3A regions.

**Figure 2:**
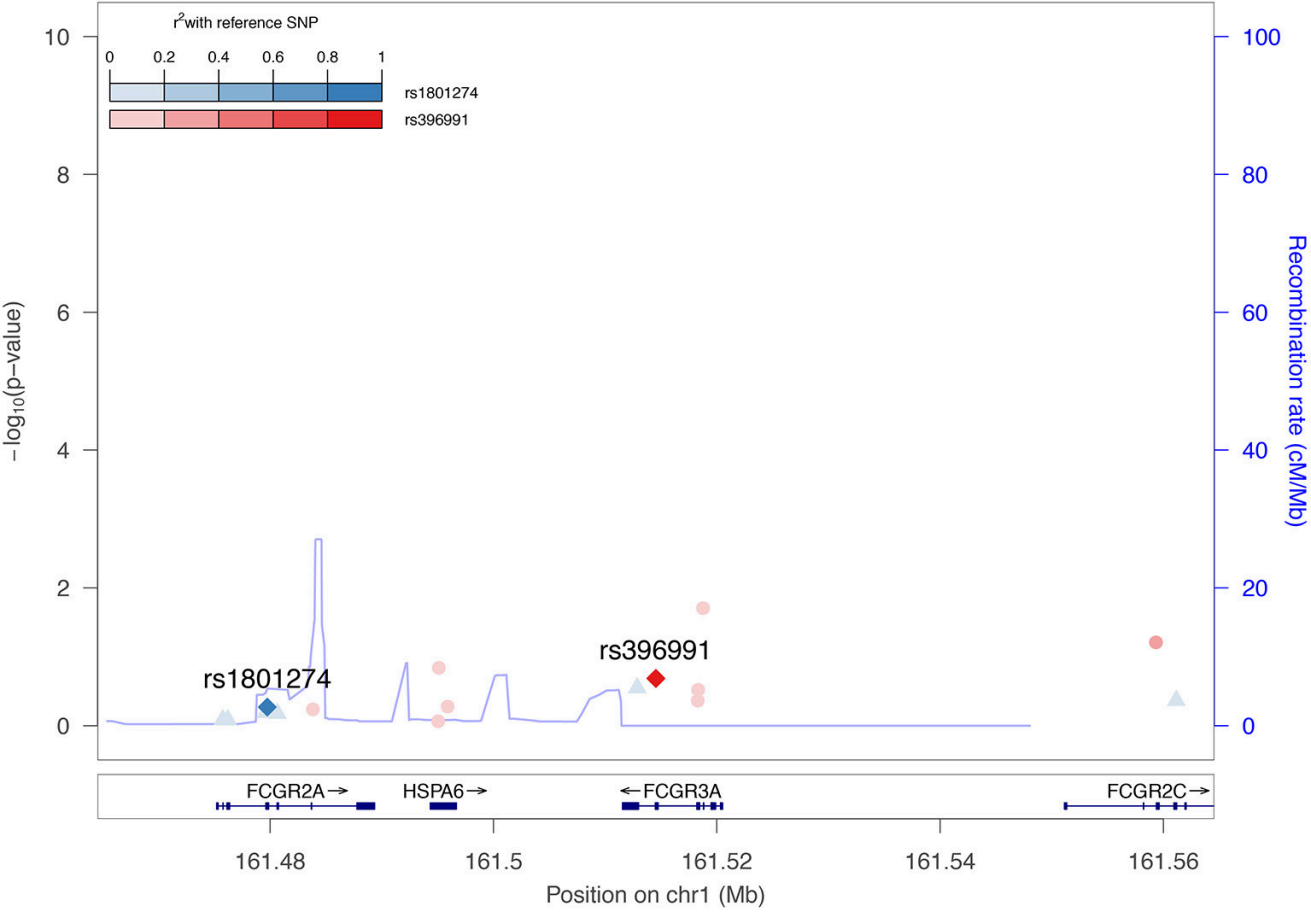
No association between FCGR2A or FCGR3A polymorphisms on HIV-1 set point viral load. Regional association plot highlighting the association between the FCGR2A (rs1801274) and FCGR3A (rs396991) polymorphisms and HIV-1 spVL across 395 exome sequenced patients (48). Color intensities represent the linkage disequilibrium (*r*^2^) of other SNPs in the region with rs1801287 and rs396991, respectively. The blue line indicates the estimated recombination rate in cM/Mb from The International HapMap Consortium (2007).

The potential associations between FCGR2A or FCGR3A variants and spVL were evaluated using a fixed-effect inverse-variance weighted meta-analysis across cohorts, including a total of 7,266 HIV positive patients of Western European ancestry. We tested all common polymorphisms (minor allele frequency >5%) in a 50 kb window around the gene. In line with previous studies, no significant association with spVL was observed. An additional analysis was performed in a subset of ICGH, consisting of 467 long-term non-progressors (individuals with CD4+ T cell counts consistently above 500 cells/mm (3) for >10 years without treatment) and 517 rapid progressors (individuals with two or more CD4+ T cell counts below 300 cells/mm (3) within 3 years after the last seronegative test result). We were unable to replicate findings by Forthal et al. and did not observe associations with HIV-1 progression (40). These analyses included the FCGR2A rs1801274 polymorphism; however, the FCGR3A polymorphism rs396991 was not available from the ICGH meta-analysis as it is not directly genotyped on most genotyping arrays and could not be reliably imputed. Thus, in order to evaluate its association with HIV-1 spVL, we reassessed a standard genome-wide association study (GWAS) using exome sequencing data from 395 individuals of European descent in the Swiss HIV Cohort Study (SHCS), following the procedures described in McLaren et al. (48). This analysis did not show any association between rs396991 and HIV-1 spVL (*p* = 0.21). Additionally, the rs1801274 did not show any association with spVL (*p* = 0.54) in the same cohort (Figure 2). The inability to replicate previous findings could be attributed to differences in sample size, clinical definition and statistical rigor employed. Globally, these new analyses confirm the previous findings that common human genetic variants in FCGR2A or FCGR3A are not associated with spontaneous control of HIV-1 infection.

### Influence of FCGR Diversity on HIV-1 Acquisition

There are no conclusive studies reporting associations of FCGR polymorphisms with HIV-1 acquisition. Two independent mother-to-child transmission cohorts reported contrasting findings of FCGR2A genotypes associating with increased infection risk in children with the high-affinity HH (rs1801274) genotype (49, 50).

Here again, we evaluated potential associations between FCGR2A and FCGR3A variants and susceptibility to HIV-1 infection by accessing the results of a previous GWAS of HIV-1 acquisition that compared 6,300 HIV-1 infected individuals and 7,200 controls of European ancestry (46). The rs1801274 polymorphism did not show any sign of association with HIV-1 acquisition (*p* = 0.81), and all other tested polymorphisms in a 50 kb window around both FCGR2A and FCGR3A were also non-significant after correction for multiple testing. The FCGR3A SNP (rs396991) was not included on the genotyping chip and could not be reliably imputed and so was not tested directly. This analysis in the largest acquisition cohort published to date adds substantial evidence to the lack of involvement of common FCGR2A and FCGR3A polymorphisms in HIV-1 acquisition.

#### Role of FCGR Polymorphisms on Outcomes After ART Initiation

**Figure 3:**
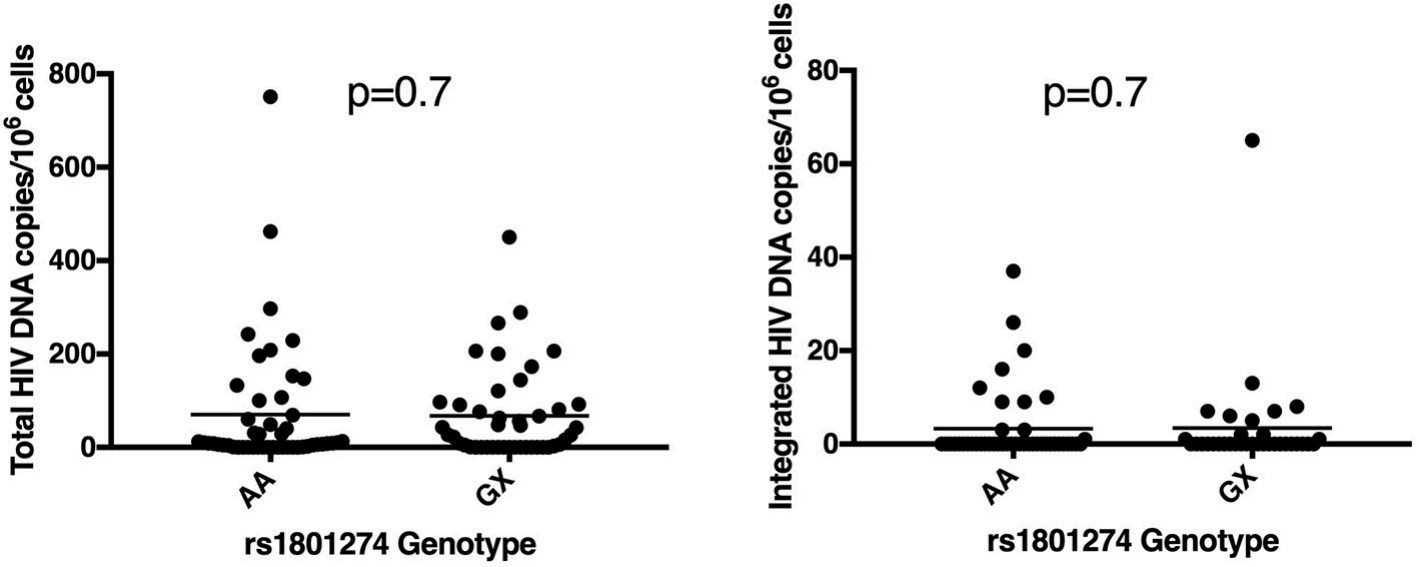
Polymorphism in FCGR2A (CD32a) does not associate with reservoir size. The rs1801274 SNP variant did not associate with levels of total or integrated HIV DNA, determined in *N* = 93 and *N* = 78 of the patients, respectively (55, 56).

Variation in genotype and expression of host genes is well established to impact HIV-1 susceptibility and disease progression in ART- naïve individuals (47). Initiation of ART in acute HIV-1 infection can limit establishment of viral reservoirs and induces post-treatment control in some individuals (51, 52). Host variation that influences viral reservoir size or reactivation during ART has not been definitively studied and has potential to significantly advance HIV cure research. There is at least one report indicating that broadly neutralizing antibodies (bNAbs) can interfere with establishment of a silent reservoir by Fc-FcR mediated mechanisms in humanized mice when administered early in the infection (53). Recently, Descours et al. identified CD32a (FCGR2A) as a marker of latently infected CD4 T cells (54). Given the previous associations of FCGR2A with HIV-1 disease pathogenesis, we hypothesized that polymorphisms in this gene might affect the size of the viral reservoir in patients that went on ART early in acute infection (55, 56). We examined genetic variation in the FCGR2A gene, characterizing polymorphisms in 436 ART-suppressed patients from the RV254 cohort. We screened for 18 variants in the extracellular domains of FCGR2A including rs1801274 and did not find associations with total or integrated HIV DNA (*p* > 0.05) (Figure 3). Surprisingly, recent reports from several independent groups confirm that they were unable to replicate the original findings from the Descours et al. study, showing that associations with post-ART control continue to be elusive (57–60).

### Effect of FCGR Variation on HIV-1 Vaccine Efficacy

Variation in host genes can impact vaccine outcomes, and other than HLA, the only other gene to impact HIV-1 vaccine efficacy was in the FCGR locus (61–63). Two studies of HIV-1 vaccine efficacy revealed a remarkable coincidence of FCGR polymorphism associated with opposing directions for efficacy, suggesting that the effect of FCGR genetic variation may be specific to vaccine regimens. The FCGR2C association study of Li et al. (62), used a direct sequencing approach to identify FCGR2C SNPs that associated with vaccine efficacy (VE) against HIV-1 in the RV144 vaccine trial, that showed modest efficacy (64). Individuals with at least one minor allele of three FCGR2C SNPs (rs114945036, rs138747765, and rs78603008) had a vaccine efficacy of 64% against any HIV-1 subtype and 91% against the CRF01-AE subtype with the protective 169K HIV-1 variant identified previously by sieve analysis (65). Although the functional mechanisms underlying the association were not revealed in this study, a subsequent examination of the FCGR2C SNPs showed rs114945036 correlated with expression levels of FCGR2A/C (66). This effect was found across different populations and was specific to the rs114945036 SNP located in the intron. Further, rs114945036 also associated with the expression of the Fc receptor-like A (FCRLA) gene, an FCGR related gene located within a gene cluster adjacent to FCGR (see Figure 1A). These results suggest that the FCGR expression is either influenced by this SNP through an undefined mechanism, or is in linkage with other causal variants that directly affect expression levels.

In the second study, four FCGR2C SNPs significantly modified the hazard ratio in the HVTN505 trial that did not show protection against HIV-1 acquisition (62). Three of the SNPs were common with those previously identified in RV144. In contrast to the RV144 study, in HVTN505 among the recipients carrying the FCGR2C minor alleles, HIV-1 acquisition risk was higher in the vaccine group than in the placebo group, in precisely the opposite direction of that observed in RV144 (efficacy against HIV-1 acquisition hazard ratios (HR) of 9.79 (*p* = 0.035) and 0.36 (*p* = 0.04), respectively). It is not clear how polymorphisms in a pseudogene functions during HIV-1 vaccination and their associations with FCGR expression may provide a novel avenue for further investigation.

Two additional studies of outcomes in Vax004, a trial testing recombinant gp120 vaccination in preventing sexually acquired HIV infection, also implicated FCGR variation in HIV infection and vaccine efficacy. Both studies tested the classical FCGR2A or FCGR3A variants comprised of the FCGR2A-R/H (rs1801274) and FCGR3A-F/V (rs396991) alleles. The first study found that lower affinity receptors (FCG2A-RR or HR and FCGR3A-FF) were associated with higher serum ADCVI activity, which itself predicted the rate of infection (67). A second study by the same group, showed the FCGR3A-VV genotype distinguished the lowest behavioral risk group from the high-risk behavioral group (68). The low risk group had a higher infection rate than low risk vaccinees with one or two F alleles (HR = 3.52; *p* = 0.002) while the high-risk group showed no association. Functional studies may be directed by these findings to interrogate quantitative and qualitative effects on FCGRs and associated antibody production. At a minimum, the intersection of these studies suggests that the impact of FCGR genetic variations on vaccine efficacy should be further investigated.

## FCGR and IGHG—Future Directions For Genetic Analysis

It is apparent that the genomic complexity of the FCGR region presents a major challenge for uncovering the underlying causal FCGR variants. Different FCGR have distinct functions and mechanisms of regulation but share highly similar sequences. While FCGR genetic variations are clearly linked to host defense against infectious diseases and other important immune functions as discussed, current approaches measure only a small portion of the existing FCGR variation. The HVTN505 and RV144 studies referenced above were by far the most comprehensive in that regard, measuring ~10 kb of the FCGR region, including functional exons encoding external protein domains and flanking intron sequences from the five FCGR genes. However, the complete FCGR region extends over 200 kb leaving open the likelihood of additional causal variation (Figure 1A). Indeed, the lack of phasing of the over 20,000 SNPs documented in the FCGR region significantly limits its direct utility for association analysis and the ultimate goal of identifying causal variants (69). Complete haplotype-resolved FCGR genomic sequences across human populations by approaches such as those used for defining variability in the KIR region may be necessary in order to provide a complete analysis of these loci (70–72).

When considering FCGR host genetics and its relationship to HIV-1 susceptibility and vaccine efficacy—or any association with disease—a natural but not often considered extension to host genetic association studies interrogating FCGR variability lies within the human immunoglobulin constant heavy G chain (IGHG) gene region on chromosomal segment 14q32.3 (73). This region encoding the human IG heavy constant genes (IGHG3, IGHG1, IGHG2, IGHA2, and the IGH locus on chromosome 14) provides access to a system for understanding immunogenicity of the polymorphic IG chains (74, 75). The evident functional relationship between FCGR and IgG constant region variability, which itself is substantial (74), argues strongly for host genetic studies of FCGR to be paired with analysis of IGHG. Although little genomic characterization for the IGHG system is available at present, we anticipate that NGS technologies will rapidly fill that void. Of course, once provided with high quality and high resolution data, significant effort will need to be invested in new sophisticated analytical approaches examining multiple factors simultaneously to find the causal variation revealing operative biological mechanisms.

## Concluding Remarks

Disease pathogenesis of HIV-1 has been shown to be modulated by allelic variants in the FCGR genes. However, such findings have not always been robust, as they were not replicated or in some cases were contradictory. There is however considerable interest in the role of Fc-mediated antiviral functions such as ADCC, ADCP, ADCD, and ADCVI in protective immunity against HIV-1 (76). Host genetics of the Fc receptors that bind to the Fc domain of the IgG antibody might modulate the functional antiviral antibody responses to HIV-1 vaccination. ADCC was previously identified as a correlate of protection in the RV144 human efficacy trial (64). More recently, ADCP has been shown to correlate with protection against acquisition of SIV/SHIV/HIV-1 in multiple preclinical and human efficacy trials (77–80). Given such associations, it would be critical to investigate association of host variation in FCGR genes and such Fc-mediated antiviral functions that are now being generated using technologies such as systems serology (1). Such findings might shed light on the role of Fc gene and receptor genotypes on HIV-1 disease pathogenesis.

## Author Contributions

RT conceptualized, organized the content of the review and wrote sections Introduction, FCGR polymorphisms and HIV-1 disease progression, Influence of FCGR diversity on HIV-1 acquisition, Role of FCGR polymorphisms on outcomes after ART initiation, and Concluding Remarks. DG contributed to sections Fcγ receptor genetic diversity, Effect of FCGR variation on HIV-1 vaccine efficacy, and FCGR and IGHG–future directions for genetic analysis. JF and CT drafted sections FCGR polymorphisms and HIV-1 disease progression, Influence of FCGR diversity on HIV-1 acquisition. All authors participated in editing and revising the manuscript.

### Conflict of Interest Statement

The authors declare that the research was conducted in the absence of any commercial or financial relationships that could be construed as a potential conflict of interest.

## References

[1] Chung AW Alter G. Systems serology: profiling vaccine induced humoral immunity against HIV. Retrovirology. (2017) 14:57. 10.1186/s12977-017-0380-3 29268769 PMC5740944

[2] Forthal DN Moog C. Fc receptor-mediated antiviral antibodies. Curr Opin HIV AIDS. (2009) 4:388–93. 10.1097/COH.0b013e32832f0a89 20048702 PMC2882066

[3] Cocklin SL Schmitz JE. The role of Fc receptors in HIV infection and vaccine efficacy. Curr Opin HIV AIDS. (2014) 9:257–62. 10.1097/coh.0000000000000051 24670320 PMC4120665

[4] Bournazos S Woof JM Hart SP Dransfield I. Functional and clinical consequences of Fc receptor polymorphic and copy number variants. Clin Exp Immunol. (2009) 157:244–54. 10.1111/j.1365-2249.2009.03980.x 19604264 PMC2730850

[5] Li X Gibson AW Kimberly RP. Human FcR polymorphism and disease. Curr Top Microbiol Immunol. (2014) 382:275–302. 10.1007/978-3-319-07911-0_13. 25116105 PMC4209745

[6] Bournazos S Ravetch JV. Fcgamma receptor function and the design of vaccination strategies. Immunity. (2017) 47:224–33. 10.1016/j.immuni.2017.07.009 28813656 PMC5573140

[7] Kratochvil S McKay PF Chung AW Kent SJ Gilmour J Shattock RJ. Immunoglobulin G1 allotype influences antibody subclass distribution in response to HIV gp140 vaccination. Front Immunol. 8:1883. (2017) 10.3389/fimmu.2017.01883 29326728 PMC5742328

[8] Gupta S Gach JS Becerra JC Phan TB Pudney J Moldoveanu Z. The Neonatal Fc receptor (FcRn) enhances human immunodeficiency virus type 1 (HIV-1) transcytosis across epithelial cells. PLoS Pathog. (2013) 9:e1003776. 10.1371/journal.ppat.1003776 24278022 PMC3836734

[9] Shen S Pyo CW Vu Q Wang R Geraghty DE. The essential detail: the genetics and genomics of the primate immune response. ILAR J. (2013) 54:181–95. 10.1093/ilar/ilt043 24174441

[10] Misra MK Augusto DG Martin GM Nemat-Gorgani N Sauter J Hofmann JA. Report from the Killer-cell Immunoglobulin-like Receptors (KIR) component of the 17th International HLA and Immunogenetics Workshop. Hum Immunol. (2018) 79:825–33 10.1016/j.humimm.2018.10.003 30321631 PMC6322681

[11] Bruhns P. Properties of mouse and human IgG receptors and their contribution to disease models. Blood. (2012) 119:5640–9. 10.1182/blood-2012-01-380121 22535666

[12] Bruhns P Iannascoli B England P Mancardi DA Fernandez N Jorieux S. Specificity and affinity of human Fcgamma receptors and their polymorphic variants for human IgG subclasses. Blood. (2009) 113:3716–25. 10.1182/blood-2008-09-179754 19018092

[13] Warmerdam PA van de Winkel JG Gosselin EJ Capel PJ. Molecular basis for a polymorphism of human Fc gamma receptor II (CD32). J Exp Med. (1990) 172:19–25. 2141627 10.1084/jem.172.1.19 PMC2188138

[14] Ravetch JV Perussia B. Alternative membrane forms of Fc gamma RIII(CD16) on human natural killer cells and neutrophils. Cell type-specific expression of two genes that differ in single nucleotide substitutions. J Exp Med. (1989) 170:481–97. 2526846 10.1084/jem.170.2.481 PMC2189395

[15] Seret G Hanrotel C Bendaoud B Le Meur Y Renaudineau Y. Homozygous FCGR3A-158F mutation is associated with delayed B-cell depletion following rituximab but with preserved efficacy in a patient with refractory lupus nephritis. Clini Kidney J. (2013) 6:74–6. 10.1093/ckj/sfs162 27818754 PMC5094396

[16] Shimizu Tanaka Y Tazawa H Verma S Onoe T Ishiyama K. Fc-gamma receptor polymorphisms predispose patients to infectious complications after liver transplantation. Am J Transplant. (2016) 16:625–33. 10.1111/ajt.13492 26517570

[17] Taylor RJ Saloura V Jain A Goloubeva O Wong S Kronsberg S. *Ex vivo* antibody-dependent cellular cytotoxicity inducibility predicts efficacy of cetuximab. Cancer Immunol Res. (2015) 3:567–74. 10.1158/2326-6066.cir-14-0188 25769300 PMC4681575

[18] Flinsenberg TW Janssen WJ Herczenik E Boross P Nederend M Jongeneel LH. A novel FcgammaRIIa Q27W gene variant is associated with common variable immune deficiency through defective FcgammaRIIa downstream signaling. Clin Immunol. (2014) 155:108–17. 10.1016/j.clim.2014.09.006 25242138

[19] Kono H Kyogoku C Suzuki T Tsuchiya N Honda H Yamamoto K. FcgammaRIIB Ile232Thr transmembrane polymorphism associated with human systemic lupus erythematosus decreases affinity to lipid rafts and attenuates inhibitory effects on B cell receptor signaling. Hum Mol Genet. (2005) 14:2881–92. 10.1093/hmg/ddi320 16115811

[20] Ernst LK Metes D Herberman RB Morel PA. Allelic polymorphisms in the FcgammaRIIC gene can influence its function on normal human natural killer cells. J Mol Med. (2002) 80:248–57. 10.1007/s00109-001-0294-2 11976734

[21] Metes D Ernst LK Chambers WH Sulica A Herberman RB Morel PA. Expression of functional CD32 molecules on human NK cells is determined by an allelic polymorphism of the FcgammaRIIC gene. Blood. (1998) 91:2369–80. 9516136

[22] Nagelkerke SQ Tacke CE Breunis WB Geissler J Sins JW Appelhof B. Nonallelic homologous recombination of the FCGR2/3 locus results in copy number variation and novel chimeric FCGR2 genes with aberrant functional expression. Genes Immun. (2015) 16:422–9. 10.1038/gene.2015.25 26133275

[23] van der Heijden J Breunis WB Geissler J de Boer M van den Berg TK Kuijpers TW Phenotypic variation in IgG receptors by nonclassical FCGR2C alleles. J Immunol. (2012) 188:1318–24. 10.4049/jimmunol.1003945 22198951

[24] Metes D Galatiuc C Moldovan I Morel PA Chambers WH DeLeo AB. Expression and function of Fc gamma RII on human natural killer cells. Nat Immun. (1994) 13:289–300. 7894200

[25] Qiu WQ de Bruin D Brownstein BH Pearse R Ravetch JV. Organization of the human and mouse low-affinity Fc gamma R genes: duplication and recombination. Science. (1990) 248:732–5. 2139735 10.1126/science.2139735

[26] de Haas M Koene HR Kleijer M de Vries E Simsek S van Tol MJ A triallelic Fc gamma receptor type IIIA polymorphism influences the binding of human IgG by NK cell Fc gamma RIIIa. J Immunol. (1996) 156:2948–55. 8609432

[27] Ory PA Clark MR Kwoh EE Clarkson SB Goldstein IM. Sequences of complementary DNAs that encode the NA1 and NA2 forms of Fc receptor III on human neutrophils. J Clin Invest. (1989) 84:1688–91. 10.1172/jci114350 2478590 PMC304039

[28] Ory PA Goldstein IM Kwoh EE Clarkson SB. Characterization of polymorphic forms of Fc receptor III on human neutrophils. J Clin Invest. (1989) 83:1676–81. 10.1172/jci114067 2523415 PMC303876

[29] Bredius RG Fijen CA De Haas M Kuijper EJ Weening RS Van de Winkel JG. Role of neutrophil Fc gamma RIIa (CD32) and Fc gamma RIIIb (CD16) polymorphic forms in phagocytosis of human IgG1- and IgG3-opsonized bacteria and erythrocytes. Immunology. (1994) 83:624–30. 7875742 PMC1415059

[30] Salmon JE Edberg JC Kimberly RP. Fc gamma receptor III on human neutrophils. Allelic variants have functionally distinct capacities. J Clin Invest. (1990) 85:1287–95. 10.1172/jci114566 1690757 PMC296565

[31] Bux J Stein EL Bierling P Fromont P Clay M Stroncek D. Characterization of a new alloantigen (SH) on the human neutrophil Fc gamma receptor IIIb. Blood. (1997) 89:1027–34. 9028335

[32] Flesch BK Doose S Siebert R Ntambi E Neppert J. FCGR3 variants and expression of human neutrophil antigen-1a,−1b, and−1c in the populations of northern Germany and Uganda. Transfusion. (2002) 42:469–75. 10.1046/j.1525-1438.2002.00087.x 12076295

[33] Reil A Sachs UJ Siahanidou T Flesch BK Bux J. HNA-1d: a new human neutrophil antigen located on Fcgamma receptor IIIb associated with neonatal immune neutropenia. Transfusion. (2013) 53:2145–51. 10.1111/trf.12086 23347194

[34] Hollox EJ Hoh BP. Human gene copy number variation and infectious disease. Human Genet. (2014) 133:1217–33. 10.1007/s00439-014-1457-x 25110110

[35] Breunis WB van Mirre E Geissler J Laddach N Wolbink G van der Schoot E Copy number variation at the FCGR locus includes FCGR3A, FCGR2C, and FCGR3B but not FCGR2A and FCGR2B. Human Mutat. (2009) 30:E640–50. 10.1002/humu.20997 19309690

[36] Niederer HA Willcocks LC Rayner TF Yang W Lau YL Williams TN. Copy number, linkage disequilibrium and disease association in the FCGR locus. Hum Mol Genet. (2010) 19:3282–94. 10.1093/hmg/ddq216 20508037 PMC2908468

[37] Hargreaves CE Rose-Zerilli MJ Machado LR Iriyama C Hollox EJ Cragg MS. Fcgamma receptors: genetic variation, function, and disease. Immunol Rev. (2015) 268:6–24. 10.1111/imr.12341 26497510

[38] Machado LR Hardwick RJ Bowdrey J Bogle H Knowles TJ Sironi M. Evolutionary history of copy-number-variable locus for the low-affinity Fcgamma receptor: mutation rate, autoimmune disease, and the legacy of helminth infection. Am J Hum Genet. (2012) 90:973–85. 10.1016/j.ajhg.2012.04.018 22608500 PMC3370267

[39] Lehrnbecher TL Foster CB Zhu S Venzon D Steinberg SM Wyvill K. Variant genotypes of FcgammaRIIIA influence the development of Kaposi's sarcoma in HIV-infected men. Blood. (2000) 95:2386–90. 10733511

[40] Forthal DN Landucci G Bream J Jacobson LP Phan TB Montoya B. FcgammaRIIa genotype predicts progression of HIV infection. J Immunol. (2007) 179:7916–23. 10.4049/jimmunol.179.11.7916 18025239

[41] Weis JF McClelland RS Jaoko W Mandaliya KN Overbaugh J Graham SM Short communication: Fc gamma receptors IIa and IIIa genetic polymorphisms do not predict HIV-1 disease progression in Kenyan women. AIDS Res Hum Retroviruses. (2015) 31:288–92. 10.1089/AID.2014.0209 25312792 PMC4348085

[42] Poonia B Kijak GH Pauza CD. High affinity allele for the gene of FCGR3A is risk factor for HIV infection and progression. PLoS ONE. (2010) 5:e15562. 10.1371/journal.pone.0015562 21187939 PMC3004964

[43] Rohatgi S Gohil S Kuniholm MH Schultz H Dufaud C Armour KL. Fc gamma receptor 3A polymorphism and risk for HIV-associated cryptococcal disease. MBio. (2013) 4:e00573–13. 10.1128/mBio.00573-13 23982074 PMC3760251

[44] Meletiadis J Walsh TJ Choi EH Pappas PG Ennis D Douglas J. Study of common functional genetic polymorphisms of FCGR2A, 3A and 3B genes and the risk for cryptococcosis in HIV-uninfected patients. Med Mycol. (2007) 45:513–8. 10.1080/13693780701390140 17710620

[45] Little J Higgins JP Ioannidis JP Moher D Gagnon F von Elm E. STrengthening the REporting of Genetic Association Studies (STREGA): an extension of the STROBE statement. PLoS Med. (2009) 6:e22. 10.1371/journal.pmed.1000022 19192942 PMC2634792

[46] McLaren PJ Coulonges C Ripke S van den Berg L Buchbinder S Carrington M. Association study of common genetic variants and HIV-1 acquisition in 6,300 infected cases and 7,200 controls. PLoS Pathog. (2013) 9:e1003515. 10.1371/journal.ppat.1003515 23935489 PMC3723635

[47] McLaren PJ Carrington M. The impact of host genetic variation on infection with HIV-1. Nat Immunol. (2015) 16:577–83. 10.1038/ni.3147 25988890 PMC6296468

[48] McLaren PJ Pulit SL Gurdasani D Bartha I Shea PR Pomilla C. Evaluating the impact of functional genetic variation on HIV-1 control. J Infect Dis. (2017) 216:1063–9. 10.1093/infdis/jix470 28968755 PMC5853944

[49] Brouwer KC Lal RB Mirel LB Yang C van Eijk AM Ayisi J. Polymorphism of Fc receptor IIa for IgG in infants is associated with susceptibility to perinatal HIV-1 infection. AIDS. (2004) 18:1187–94. 10.1097/00002030-200405210-00012 15166534

[50] Milligan C Richardson BA John-Stewart G Nduati R Overbaugh J. FCGR2A and FCGR3A genotypes in human immunodeficiency virus mother-to-child transmission. Open Forum Infect Dis. (2015) 2:ofv149. 10.1093/ofid/ofv149 26613093 PMC4653957

[51] Ananworanich J Chomont N Eller LA Kroon E Tovanabutra S Bose M. HIV DNA set point is rapidly established in acute HIV infection and dramatically reduced by early ART. EBioMedicine. (2016) 11:68–72. 10.1016/j.ebiom.2016.07.024 27460436 PMC5049918

[52] Saez-Cirion A Bacchus C Hocqueloux L Avettand-Fenoel V Girault I Lecuroux C. Post-treatment HIV-1 controllers with a long-term virological remission after the interruption of early initiated antiretroviral therapy ANRS VISCONTI Study. PLoS Pathog. (2013) 9:e1003211. 10.1371/journal.ppat.1003211 23516360 PMC3597518

[53] Halper-Stromberg A Lu CL Klein F Horwitz JA Bournazos S Nogueira L. Broadly neutralizing antibodies and viral inducers decrease rebound from HIV-1 latent reservoirs in humanized mice. Cell. (2014) 158:989–99. 10.1016/j.cell.2014.07.043 25131989 PMC4163911

[54] Descours B Petitjean G López-Zaragoza JL Bruel T Raffel R Psomas C. CD32a is a marker of a CD4 T-cell HIV reservoir harbouring replication-competent proviruses. Nature. (2017) 543:564–7. 10.1038/nature21710 28297712

[55] De Souza MS Phanuphak N Pinyakorn S Trichavaroj R Pattanachaiwit S Chomchey N Impact of nucleic acid testing relative to antigen/antibody combination immunoassay on the detection of acute HIV infection. AIDS. (2015) 29:793–800. 10.1097/QAD.0000000000000616 25985402

[56] Ananworanich J Fletcher JL Pinyakorn S van Griensven F Vandergeeten C Schuetz A. A novel acute HIV infection staging system based on 4th generation immunoassay. Retrovirology. (2013) 10:56. 10.1186/1742-4690-10-56 23718762 PMC3669623

[57] Abdel-Mohsen M Kuri-Cervantes L Grau-Exposito J Spivak AM Nell RA Tomescu C CD32 is expressed on cells with transcriptionally active HIV but does not enrich for HIV DNA in resting T cells. Sci Transl Med. (2018) 10:eaar6759 10.1126/scitranslmed.aar6759 29669853 PMC6282755

[58] Osuna CE Lim SY Kublin JL Apps R Chen E Mota TM Evidence that CD32a does not mark the HIV-1 latent reservoir. Nature. (2018) 561:E20–8. 10.1038/s41586-018-0495-2 30232424 PMC6528470

[59] Bertagnolli LN White JA Simonetti FR Beg SA Lai J Tomescu C. The role of CD32 during HIV-1 infection. Nature. (2018) 561:E17–9. 10.1038/s41586-018-0494-3 30232425 PMC6442722

[60] Perez L Anderson J Chipman J Thorkelson A Chun TW Moir S. Conflicting evidence for HIV enrichment in CD32(+) CD4 T cells. Nature. (2018) 561, E9–16. 10.1038/s41586-018-0493-4 30232423 PMC6410373

[61] Prentice HA Tomaras GD Geraghty DE Apps R Fong Y Ehrenberg PK. HLA class II genes modulate vaccine-induced antibody responses to affect HIV-1 acquisition. Sci Transl Med. (2015) 7:296ra112. 10.1126/scitranslmed.aab4005 26180102 PMC4911012

[62] Li SS Gilbert PB Tomaras GD Kijak G Ferrari G Thomas R. FCGR2C polymorphisms associate with HIV-1 vaccine protection in RV144 trial. J Clin Invest. (2014) 124:3879–90. 10.1172/JCI75539 25105367 PMC4151214

[63] Gartland AJ Li S McNevin J Tomaras GD Gottardo R Janes H. Analysis of HLA A^*^02 association with vaccine efficacy in the RV144 HIV-1 vaccine trial. J Virol. (2014) 88:8242–55. 10.1128/JVI.01164-14 24829343 PMC4135964

[64] Rerks-Ngarm S Pitisuttithum P Nitayaphan S Kaewkungwal J Chiu J Paris R. Vaccination with ALVAC and AIDSVAX to prevent HIV-1 infection in Thailand. N Engl J Med. (2009) 361:2209–20. 10.1056/NEJMoa0908492 19843557

[65] Rolland M Edlefsen PT Larsen BB Tovanabutra S Sanders-Buell E Hertz T. Increased HIV-1 vaccine efficacy against viruses with genetic signatures in Env V2. Nature. (2012) 490:417–20. 10.1038/nature11519 22960785 PMC3551291

[66] Peng X Li SS Gilbert PB Geraghty DE Katze MG. FCGR2C polymorphisms associated with HIV-1 vaccine protection are linked to altered gene expression of Fc-gamma receptors in human B cells. PLoS ONE. (2016) 11:e0152425. 10.1371/journal.pone.0152425 27015273 PMC4807760

[67] Forthal DN Gilbert PB Landucci G Phan T. Recombinant gp120 vaccine-induced antibodies inhibit clinical strains of HIV-1 in the presence of Fc receptor-bearing effector cells and correlate inversely with HIV infection rate. J Immunol. (2007) 178:6596–603. 17475891 10.4049/jimmunol.178.10.6596

[68] Forthal DN Gabriel EE Wang A Landucci G Phan TB. Association of Fcgamma receptor IIIa genotype with the rate of HIV infection after gp120 vaccination. Blood. (2012) 120:2836–42. 10.1182/blood-2012-05-431361 22915639 PMC3466964

[69] EMBL-EBI Ensembl Variation Database. (2018). Avaliable online at: https://www.ensembl.org/info/genome/variation/index.html

[70] Pyo CW Guethlein LA Vu Q Wang R Abi-Rached L Norman PJ. Different patterns of evolution in the centromeric and telomeric regions of group A and B haplotypes of the human killer cell Ig-like receptor locus. PLoS ONE. (2010) 5:e15115. 10.1371/journal.pone.0015115 21206914 PMC3012066

[71] Pyo CW Wang R Vu Q Cereb N Yang SY Duh FM. Recombinant structures expand and contract inter and intragenic diversification at the KIR locus. BMC Genomics. (2013) 14:89. 10.1186/1471-2164-14-89 23394822 PMC3606631

[72] Roe D Vierra-Green C Pyo CW Eng K Hall R Kuang R. Revealing complete complex KIR haplotypes phased by long-read sequencing technology. Genes Immun. (2017) 18:127–34. 10.1038/gene.2017.10 28569259 PMC5637231

[73] Oxelius VA Pandey JP. Human immunoglobulin constant heavy G chain (IGHG) (Fcgamma) (GM) genes, defining innate variants of IgG molecules and B cells, have impact on disease and therapy. Clin Immunol. (2013) 149:475–86. 10.1016/j.clim.2013.10.003 24239836

[74] Lefranc MP Lefranc G. Human Gm, Km, and Am allotypes and their molecular characterization: a remarkable demonstration of polymorphism. Methods Mol Biol. (2012) 882:635–80. 10.1007/978-1-61779-842-9_34 22665258

[75] Pandey JP Li Z. The forgotten tale of immunoglobulin allotypes in cancer risk and treatment. Exper Hematol Oncol. (2013) 2:6. 10.1186/2162-3619-2-6 23425356 PMC3598368

[76] Lewis GK. Role of Fc-mediated antibody function in protective immunity against HIV-1. Immunology. (2014) 142:46–57. 10.1111/imm.12232 24843871 PMC3992047

[77] Barouch DH Alter G Broge T Linde C Ackerman ME Brown EP. Protective efficacy of adenovirus/protein vaccines against SIV challenges in rhesus monkeys. Science. (2015) 349:320–4. 10.1126/science.aab3886 26138104 PMC4653134

[78] Barouch DH Tomaka FL Wegmann F Stieh DJ Alter G Robb ML. Evaluation of a mosaic HIV-1 vaccine in a multicentre, randomised, double-blind, placebo-controlled, phase 1/2a clinical trial (APPROACH) and in rhesus monkeys (NHP 13-19). Lancet. (2018) 392:232–43. 10.1016/S0140-6736(18)31364-3 30047376 PMC6192527

[79] Barouch DH Stephenson KE Borducchi EN Smith K Stanley K McNally AG. Protective efficacy of a global HIV-1 mosaic vaccine against heterologous SHIV challenges in rhesus monkeys. Cell. (2013) 155:531–9. 10.1016/j.cell.2013.09.061 24243013 PMC3846288

[80] Issac B Ehrenberg PK Eller M Alter G Sekaly RP Robb ML Vaccine-induced gene signature correlates with protection against acquisition in three independent vaccine efficacy trials including RV144. In: Conference Abstract. HIV Research for Prevention HIVR4P. Madrid (2018).

